# A reproducible reference point for the common peroneal nerve during surgery at the posterolateral corner of the knee: a cadaveric study

**DOI:** 10.1186/s43019-020-00039-2

**Published:** 2020-05-24

**Authors:** Hong Lee Terry Teo, Ke Xin Magneline Ang, Sir Young James Loh

**Affiliations:** Department of Orthopaedic Surgery, Changi General Hospital, Singapore, Singapore

**Keywords:** Common peroneal nerve injury, Posterolateral corner, Reference point, Fibular head, Fibular neck, Biceps femoris

## Abstract

**Background:**

This is an experimental study conducted to assess whether the fibular head is a reliable reference point to identify the position of the common peroneal nerve at the posterolateral corner of the knee.

**Materials and methods:**

Twelve cadaveric knees were dissected through the lateral approach. The common peroneal nerve was identified and traced. The location where the common peroneal nerve crossed the posterior border of the biceps femoris and the posterior border of the fibular neck were designated as points B and N, respectively. The tip of the fibular head was designated F. Distances FB and FN were measured and the triangular area FBN was calculated at various degrees of knee flexion.

**Results:**

During knee motion, distance FN showed minimal change and was not affected by variation in degrees of knee flexion (*p* = 0.131). Distance FB and distance BN were affected by variation in degrees of knee flexion (*p* < 0.001). Triangular area FBN increased in size up to 60° of knee flexion measuring 621.22 mm^2^ and subsequently decreased with further knee flexion.

**Conclusion:**

The common peroneal nerve can consistently be found at approximately 20.7 ± 1 mm on the fibular neck with respect to the tip of the fibular head. The tip of the fibular head is a consistent landmark that can be used to predict the position of the exit point of the common peroneal nerve at the posterolateral corner of the knee.

## Introduction

The common peroneal nerve (CPN) is the main neurological structure present at the posterolateral corner (PLC) of the knee joint and it is the most important structure passing through this location. It is one of the two nerves arising from the bifurcation of the sciatic nerve in the posterior aspect of the thigh and is supplied by branches of L4–S2 spinal nerve roots. It travels along the biceps femoris and around the neck of the fibula, and splits into the superficial and deep peroneal nerves. It innervates the muscles of the anterior and lateral compartments of the leg, which play an important role in gait. The purpose of this experimental study is to assess whether the fibular head is a reliable reference point to identify the position of the CPN at the PLC of the knee.

The CPN is particularly susceptible to injury because of its fixed attachment in the region of the neck of the fibula. In PLC injuries [1, 2] when the knee is subjected to varus and hyperextension forces, there may be traction injury of the CPN alongside alteration of the anatomy of the PLC. Recovery is generally considered to be poor [3, 4]. CPN neuropraxia has been reported due to hematoma formation at the fibular head after primary injury [5] and may also be a concern should a post-operative hematoma lead to nerve compression.

During surgeries at the PLC, the CPN is potentially at risk and the devastating consequences of injury to this structure cannot be over emphasised. CPN neurolysis is typically performed during procedures for PLC to minimise the risk of foot drop post-operatively due to swelling. Risk of CPN palsy post-operatively can be as high as 2.5% from various large retrospective meta-analyses performed [1]. Such a risk can be controlled with an easily reproducible and relatively precise guide to the relation of the nerve with surrounding identifiable landmarks. The authors hypothesise that the tip of the fibular head is a reliable reference point to predict the position of the CPN for use during surgery at the PLC.

## Materials and methods

### Specimens

This is a cadaveric study to assess the position of the CPN in relation to a reliable landmark at the PLC of the knee. It was performed under the funding of a local hospital grant (IO no. 12200257).

Twelve knee specimens from nine Caucasian cadavers were procured for this study. These consist of four male and five female cadavers with a contribution of six knee specimens from each gender. All the cadavers are from individuals who died from non-traumatic causes, aged 59 to 95 years old (mean 80.25 years). They had a height of 152.2–182.9 cm (mean 167.9 cm) and weighed 56.7–78.1 kg (mean 67.4 kg). The specimen data is provided in Table 1.

**Table 1:** Basic characteristics of specimens: anthropometric measurements of cadavers

No.	Laterality	Age (years)	Sex	Height (cm)	Weight (kg)	Body Mass Index
**1**	Left	88	Female	152.4	68.2	29
**2**	Left	59	Female	167.6	78.1	28
**3**	Right	59	Female	167.6	78.1	28
**4**	Left	85	Male	182.9	64.4	19
**5**	Left	73	Female	167.6	66.7	24
**6**	Right	85	Male	182.9	64.4	19
**7**	Left	89	Male	162.6	64.0	22.8
**8**	Left	73	Male	175.3	61.2	19.9
**9**	Left	95	Female	160.0	71.2	27.8
**10**	Right	89	Male	162.6	64.0	22.8
**11**	Right	92	Male	177.8	71.2	22.5
**12**	Right	76	Female	154.9	56.7	23.6
**Mean**	–	80.25	–	167.9	67.4	23.9
**Standard deviation**	–	12.21	–	10.13	6.42	3.61
**Variance**	–	149.11	–	102.59	41.2	13.02
**Skewness**	–	− 0.79	–	0.16	0.43	0.10
**Kurtosis**	–	−0.52	–	− 0.97	−0.15	−1.35

*BMI* Body Mass Index

The knee specimens were carefully examined and tested manually for any sign of injuries or instability. The specimens were all stored at − 20 °C and defrosted 12 to 14 h prior to examination and dissection.

### Surgical technique for anatomical examination

The study constituted careful and detailed dissection of cadaveric knee specimens. Dissection was initiated via a curvilinear incision on the lateral knee specimen in 90° of flexion. This involved starting 50 mm proximal to the lateral epicondyle and curving the incision distal to the lateral epicondyle. The incision passed between Gerdy’s tubercle and the fibular head. It concluded approximately 30 mm distal to the level of the fibular neck. Incision lengths were measured using a standard 15-cm ruler. The subcutaneous dissection was performed to the iliotibial band, then the distal biceps femoris and fibular neck were adequately exposed.

**Figure. 1:**
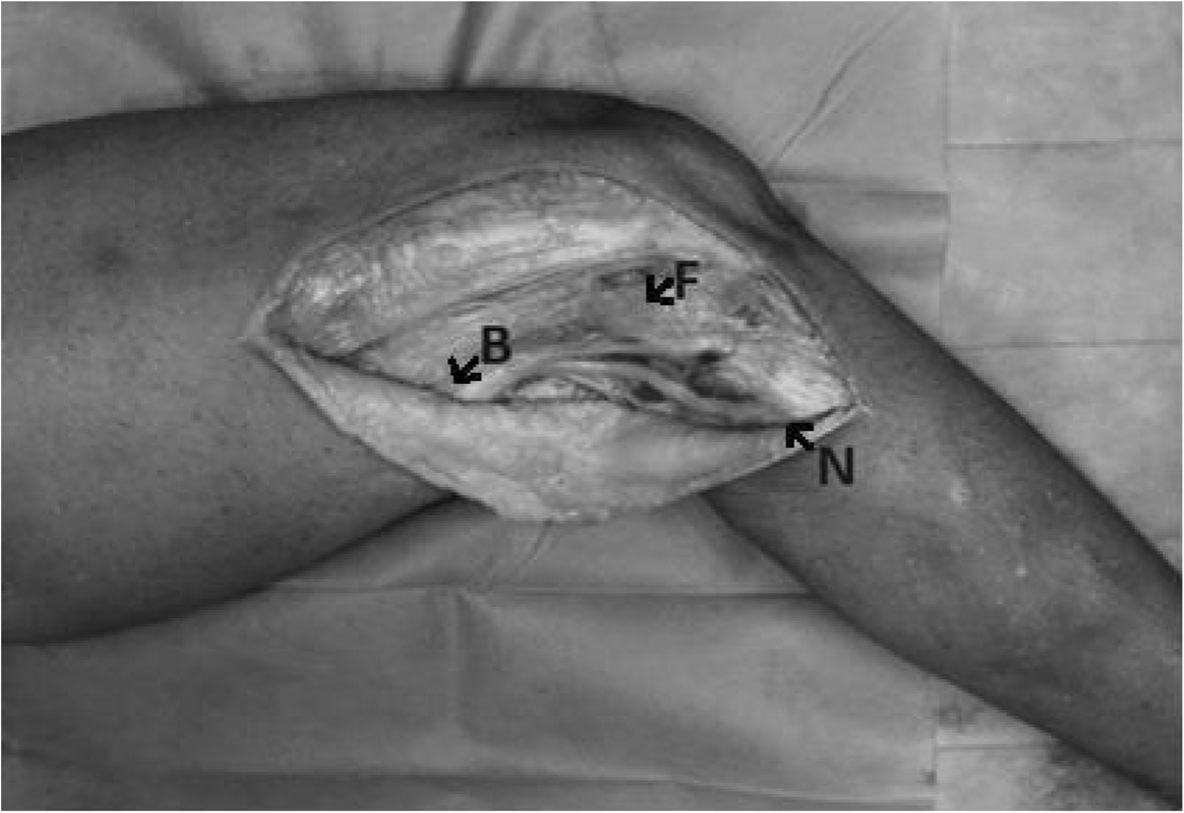
Picture of a specimen showing the incision, exposed area and points F, B and N

The CPN was next exposed only enough to identify its path. The point that it crossed the posterior border of the biceps femoris was designated B and the point that it penetrated the fascia at the level of the fibular neck was designated N. The attachments of the nerve at points B and N, as well as the soft tissue between these two points were carefully preserved. The tip of the fibular head was designated F. This was identified via a longitudinal incision over the palpable structure, adequate to identify it. Illustration 1 shows a schematic diagram of the PLC. Figure 1 is a picture of one of the specimens after dissection. The distance between the point that the CPN crosses the posterior border of the biceps femoris (B) and the tip of the fibular head (F) was designated FB. The distance between the point that the CPN enters the fascia at the level of the fibular neck (N) and the tip of the fibular head (F) was designated FN. FB and FN were measured at various degrees of knee flexion. The triangular area within the borders of FB, FN and the path of the CPN was calculated using Heron’s formula [6]. For two specimens, area FBN could not be calculated hence these two specimens were excluded during analysis for area FBN. For these two specimens, area FBN could not be calculated as the knee was placed at 0° of flexion (in full extension) and the three points F, B and N were collinear.

**Illustration 1:**
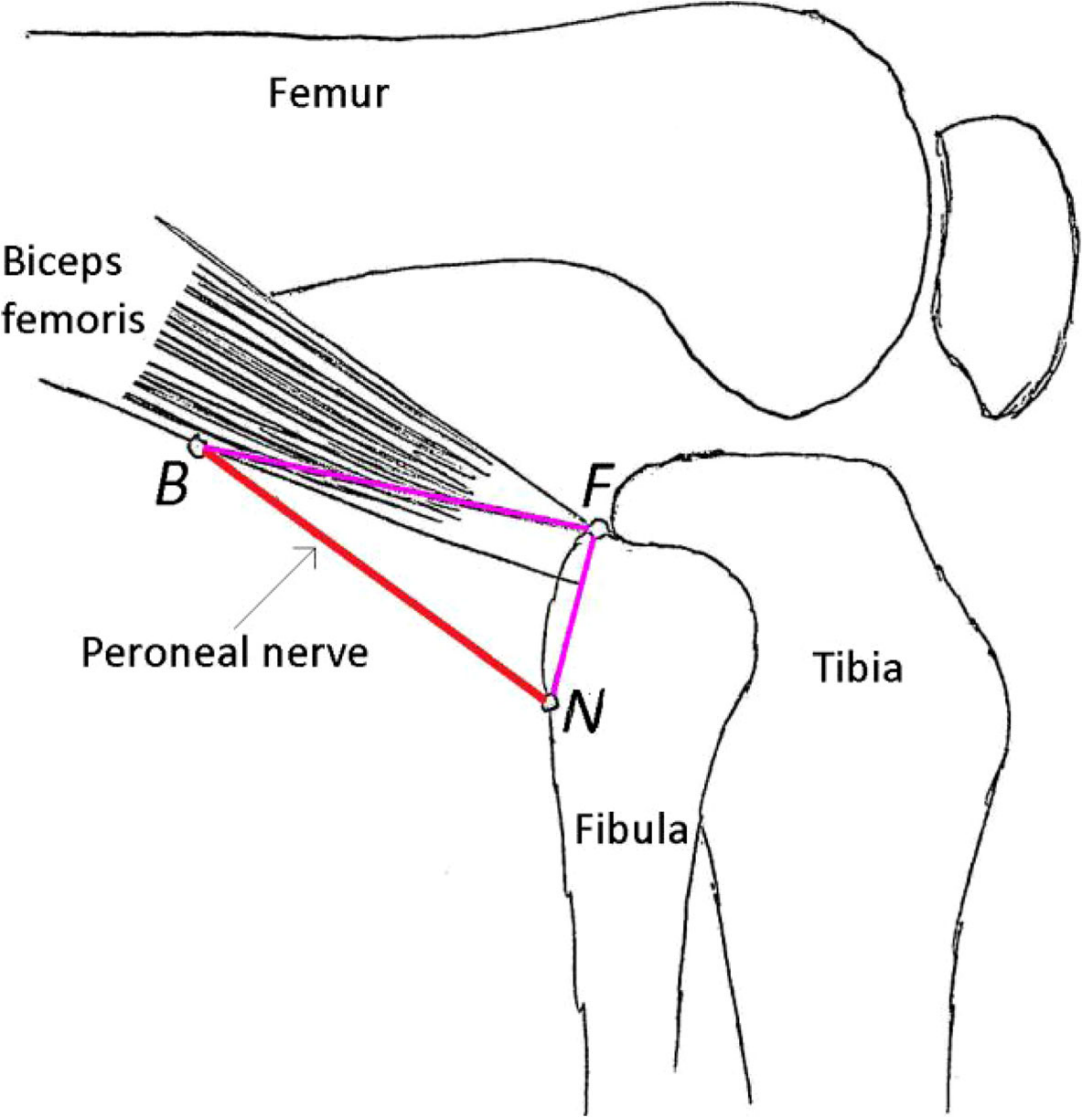
Schematic diagram: showing the tip of the fibular head (F), the posterior border of the biceps femoris where the common peroneal nerve (CPN) crosses (B) and the posterior border of the fibular neck where the CPN crosses (N)

The measurements were further replicated with the knee placed in 0, 30, 60 and 120° of flexion. An electronic goniometer was attached to the specimen to confirm the flexion angle of the specimen.

After tabulation of results, data analysis was performed using SPSS 20.0 statistical software. The means of distances FN and FB, length BN and area FBN were compared using one-way analysis of variance (ANOVA) tests. Post-hoc tests were performed using Tukey’s Honestly Significant Difference (HSD) test. A *p* value of < 0.05 was considered significant.

## Results

The CPN was identified in all 12 specimens. Mean measurements at varying angles of the various distances between the tip of the fibular head (F), the posterior border of the fibular neck where the CPN crosses (N) and the posterior border of the biceps femoris where the CPN crosses (B) are provided in Table 2. Post-hoc analysis of mean difference between subgroups is provided in Table 3.

**Table 2:** Findings of various distances from the tip of the fibular head (F), to the posterior border of the biceps femoris where the peroneal nerve crosses (B) and the posterior border of the fibular neck where the peroneal nerve crosses (N), and the area of the triangle FBN

	Degrees of knee flexion	No.	Mean (95% CI)
**Distance FN (mm)**	0	12	19.8 (17.5–22.2)
	30	12	21.7 (19.3–24.1)
	60	12	22.7 (20.0–25.4)
	90	12	20.1 (18.3–21.8)
	120	12	19.3 (17.0–21.5)
	–	60	20.7 (19.7–21.7)
**Distance FB (mm)**	0	12	57.9 (48.0–67.9)
	30	12	62.3 (55.0–69.7)
	60	12	59.5 (49.6–69.4)
	90	12	48.1 (40.5–55.7)
	120	12	37.8 (31.0–44.7)
	–	60	53.1 (49.1–57.2)
**Distance BN (mm)**	0	12	72.8 (61.6–84.0)
	30	12	76.1 (67.9–84.4)
	60	12	69.2 (59.8–78.49)
	90	12	52.2 (46.0–58.5)
	120	12	31.8 (27.2–36.4)
	–	60	60.4 (55.1–65.8)
**Area FBN (mm**^**2**^**)**	0	10	423.5 (252.9–594.1)
	30	12	530.5 (407.1–653.8)
	60	12	621.2 (468.4–774.1)
	90	12	466.6 (362.3–570.8)
	120	12	296.3 (229.8–362.8)
	–	58	469.1 (412.1–526.2)

**Table 3:** Post-hoc Tukey’s Honestly Significant Difference (HSD) for distances FN, FB, BN and area FBN (n.s. = not significant)

Difference in groups	Distance FN (mm)		Distance FB (mm)		Distance BN (mm)		Area FBN (mm^**2**^)	
	Mean difference (95% CI)	*p* value	Mean difference (95% CI)	*p* value	Mean difference (95% CI)	*p* value	Mean difference (95% CI)	*p* value
0–30	− 1.84 (− 6.02–2.34)	n.s.	− 4.42 (− 19.71–10.88)	n.s.	− 3.33 (− 18.29–11.63)	n.s.	− 107.00 (− 340.69–126.70)	n.s.
0–60	− 2.85 (− 7.03–1.33)	n.s.	− 1.57 (− 16.86–13.73)	n.s.	3.65 (− 11.31–18.61)	n.s.	− 197.75 (− 431.45–35.95)	n.s.
0–90	− 0.21 (− 4.39–3.97)	n.s.	9.85 (− 5.44–25.14)	n.s.	20.57 (5.61–35.53)	**< 0.001**	− 43.12 (− 276.81–190.59)	n.s.
0–120	0.58 (− 3.60–4.76)	n.s.	20.09 (4.80–35.38)	**0.004**	40.98 (26.02–55.93)	**< 0.001**	127.18 (− 106.52–360.88)	n.s.
30–60	− 1.01 (− 5.19–3.17)	n.s.	2.85 (− 12.44–18.14)	n.s.	6.98 (− 7.98–21.94)	n.s.	− 90.75 (− 313.58–132.07)	n.s.
30–90	1.63 (− 2.55–5.81)	n.s.	14.27 (− 1.03–29.56)	n.s.	23.90 (8.94–38.86)	**< 0.001**	63.89 (− 158.94–286.71)	n.s.
30–120	2.43 (− 1.76–6.61)	n.s.	24.51 (9.22–39.80)	**< 0.001**	44.31 (29.35–59.27)	**< 0.001**	234.18 (11.36–457.00)	**0.035**
60–90	2.64 (−1.54–6.82)	n.s.	11.42 (− 3.87–26.71)	n.s.	16.92 (1.96–31.88)	**0.019**	154.64 (− 68.18–377.46)	n.s.
60–120	3.43 (− 0.75–7.61)	n.s.	21.66 (6.37–36.95)	**0.002**	37.33 (22.37–52.28)	**< 0.001**	324.93 (102.11–547.76)	**0.001**
90–120	0.79 (− 3.39–4.97)	n.s.	10.24 (− 5.05–25.53)	n.s.	20.41 (5.45–35.37)	**0.003**	170.29 (− 52.53–393.12)	n.s.

### Distance FN

The distance FN between the tip of the fibular head and the posterior border of the fibular neck where the CPN crosses showed minimal change despite varying degrees of knee flexion from 0 to 120°. The mean value of distance FN was 20.7 mm (95% CI 19.7–21.7). The distance was longest at 60° of knee flexion where it was 22.7 mm. However, there was no difference between means of FN (*p* = 0.131) at varying degrees of knee flexion.

### Distance FB

The distance FB between tip of the fibular head and the posterior border of the biceps femoris where the CPN crosses showed an initial increase from up to 30°, at 62.34 mm, before it subsequently decreased with further knee flexion. The mean value of distance FB was 53.1 mm and it was statistically different at varying degrees of knee flexion (*p* < 0.001). Post-hoc analysis revealed significant differences in means between 0-120° (*p* = 0.004), 30-120° (*p* < 0.001) and 60-120° (*p* = 0.002).

### Distance BN

The length BN of the peroneal nerve between the two fixed points B and N was greatest at 76.1 mm at 30° of flexion. The mean value of length BN was 60.4 mm, and this was also statistically different at varying degrees of knee flexion (*p* < 0.001). Post-hoc analysis revealed significant differences in means amongst almost all groups, except between 0-30°, 0-60° and 30-60°.

### Area FBN

The triangular area FBN was largest at 60° of knee flexion at 621.2 mm^2^, respectively. The space decreased with further increasing angles of knee flexion. The mean value of area FBN was 469.1 mm^2^ (95% CI 412.1–526.2) and this was statistically different at varying degrees of knee flexion (*p* = 0.003). Post-hoc analysis revealed significant differences in means between 30-120° (*p* = 0.035) and 60-120° (*p* = 0.001).

## Discussion

An injury to the CPN potentially results in significant morbidity such as a permanent foot drop, which can occur in up to 2.5% of cases [1]. A clear and precise reference is important to the surgeon during surgery at the PLC, to reduce risks of iatrogenic damage. This is especially so if there is altered anatomy at the PLC that may make identification of the CPN even more challenging.

This study found that the mean position of the CPN at the PLC with respect to the tip of the fibular head was at 53.1 ± 4.1 mm on the biceps femoris and 20.7 ± 1 mm on the fibular neck. Hildebrand reported a value of 21.9 ± 1.8 mm from the tip of the fibular styloid to the posterior border of the fibula [7]. Of note, there was no difference in mean value with change in degree of knee flexion for position on the fibular neck (*p* = 0.131), indicating that the exit point of the CPN at the PLC could be more consistently identified regardless of knee position. Surgeons who are not familiar with this region may opt to first locate the CPN on the fibular neck and trace its course proximally given a typical anatomy with approximately 81% of the cases having division of the CPN into its deep and superficial branches at, or distal to, the fibular neck [8]. These are objective distances from the fibular head, which is a fixed reference point except in certain cases such as displaced fibular-head fractures, or proximal tibiofibular joint dislocations. These distances enable the identification and protection of the nerve during surgery and mobilisation of the nerve should the need arise. This potentially reduces the risk of nerve injury intra-operatively.

Both Hildebrand and Thi reported similar distances between the fibular head and the CPN as it enters the PLC at the biceps femoris at varying angles of knee flexion [7, 9]. However, this study did not concur, and also found that the value of distance FB is variable depending on degree of knee flexion (*p* < 0.001). As such, it is difficult to predict the entry point of the CPN into the PLC. This correlates with the more mobile attachment where the nerve crosses the posterior border of the biceps femoris, compared to the lesser mobile point at which the nerve penetrates the fascia to continue its course in the peroneal compartment. Mobility of the CPN can be improved by proximal and distal release – of more importance is the distal entry into the peroneal compartment. A fascial release at the distal entry point into the peroneal compartment during surgery would prevent unnecessary tension on the nerve during the surgery, and potentially reduce post-surgical neuropraxia due to compromise of the intrafascicular microcirculation [10].

The area demarcated by these landmarks is the working space available during surgery, and is dependent on the degree of knee flexion (*p* = 0.003). It is dynamically adjusted during surgery by positioning the knee according to the exposure required at a particular stage in surgery. It was largest at 30 and 60° of flexion, which indicates that the peroneal nerve is likely under the greatest tension in those positions. This is based on the assumption that tension is proportionately correlated with distance between the two fixed points. Thus, it is assumed that the tension on the nerve is proportionately increased when the knee is moved to 60 and 30°, respectively. Since surgery at the PLC is often performed at 90° of flexion, the CPN is not maximally stretched and, hence, risk of neuropraxia arising due to tension is not increased. Should more space and exposure be required during surgery, the angle of knee flexion could be reduced from 90° to 60° in order to optimise the area available. However, caution should still be applied when handling the CPN under greater tension in these positions as there is risk of nerve injury when intending to improve its exposure at the PLC.

### Limitations

There are several limitations in this study.

Firstly, the CPN is not completely immobile at where it crosses the posterior border of the biceps femoris, with attachments to it by the interstitial tissue. This could possibly have resulted in a lower value of FB as the nerve, with its elasticity and tensile strength (albeit limited), could be tauter during changes in knee angles from extension to 90° of flexion. Secondly, the study is an attempt, using two-dimensional (2D) measurement, to demarcate a three-dimensional (3D) zone. This could potentially be overcome by the use of non-contact measurement systems, otherwise known as photogrammetry, to capture multiple 2D images and reverse-engineer the 3D measurements from captured images [11]. Thirdly, the accuracy of the results was limited by the number of cadaveric knee specimens available. However, it should be noted that despite wide variations in anthropometric measurements of specimens that may not be representative of the normal population, there was normal variance noted for measurements FN, FB and BN. Lastly, the measurements used in this study and other literature are usually to a tenth of a millimeter, but this may be different in actual surgery as considerations must be taken in context to the patients’ size and individual anatomy. However, this study has likely obtained a close estimate to serve the purpose of aiding navigation for a surgeon.

## Conclusions

A reproducible reference to a relatively consistent landmark facilitates the identification of the CPN during surgery at the PLC. These measurements are potentially useful for a surgeon who is not familiar with this region. The study also showed that the routine working position of 90° of flexion also ensures that the CPN is not subjected to tension and an inadvertent increased risk of neuropraxia.

## Data Availability

The datasets during and/or analysed during the current study are available from the corresponding author on reasonable request.

## Abbreviations

BN: Common peroneal nerve from entry to exit point of posterolateral corner
CPN: Common peroneal nerve
FB: Tip of fibular head to the point where the common peroneal nerve crosses the posterior border of biceps femoris
FN: Tip of fibular head to the point where the common peroneal nerve enters the fascia at the level of the fibular neck
FBN: Triangular area within the borders of FB, FN and the path of the common peroneal nerve
PLC: Posterolateral corner
2D: Two-dimensional
3D: Three-dimensional
